# Trace element biomineralisation in the carapace in male and female *Argulus japonicus*

**DOI:** 10.1371/journal.pone.0197804

**Published:** 2018-06-13

**Authors:** Beric M. Gilbert, Annemariè Avenant-Oldewage

**Affiliations:** Department of Zoology, University of Johannesburg, Johannesburg, Gauteng, South Africa; Fred Hutchinson Cancer Research Center, UNITED STATES

## Abstract

Parasites of fishes have been shown to be effective bioindicators of the aquatic environment. Few investigations have been conducted on ectoparasite models and therefore little is known about the fate of trace elements and metals which they accumulate. In this study trace element sequestration was observed in the carapace of the fish louse, *Argulus japonicus* and found to relate to the sex of the parasite, as well as, the degree of sclerotization of the carapace. Adults of *A*. *japonicus* were collected from cyprinid hosts in the Vaal Dam, South Africa. Parasites were removed and flash frozen in liquid nitrogen before being sectioned with a cryomicrotome. Sections and whole mounts of parasites were prepared and treated with Phen–Green ^TM^ FL cell–permeant diacetate. Cryosections were assessed for trace elements and metals using a scanning electron microscope equipped with energy dispersive spectroscopy. Results indicated that in both male and female parasites, trace elements become bound to the carapace and produce more intense fluorescence than in soft tissues. Sexual dimorphic differences were further observed between male and female parasites. The intensity of the fluorescence signals was greater in the carapace of male parasites than in females, particularly when comparing the carapace of the ventral side of the thorax. In females, an amorphous layer of material surrounding the eggs was observed and produced an intense fluorescent signal. Levels of trace elements and metals detected were not significantly different between male and female parasites. Results observed serve as a demonstration for the first time of trace element sequestration in a freshwater crustacean parasite and possible mechanisms employed to reduce body burdens of trace elements and metals.

## Introduction

Aquatic organisms are continually exposed to a highly dynamic environment which in many instances is loaded with extraneous chemicals, most of which are of anthropogenic origin. Trace elements and metals are omnipresent components of aquatic ecosystems, generally occurring in low concentrations. Aquatic organisms are therefore constantly exposed to and accumulate these elements in their bodies. Studies investigating accumulation of trace elements and metals in aquatic invertebrates have found this to be highly variable between different taxa and even in individuals within the same taxon [1]. As a result of the deleterious effects of exposure to toxic elements and high levels of essential elements, organisms have developed mechanisms to effectively manage and reduce body burdens. In most cases, this entails the binding of trace elements to proteins and inert tissues as a means of detoxification. Several fates exist for metals accumulated by invertebrates [2]. In crustaceans, this has been found to involve the gastrointestinal tract, haemocytes and antennal glands [3], as well as, the carapace [4]. The crustacean carapace is principally composed of highly organised α-chitin chains arranged in helicoid bundles, resembling a honey comb [5]. The exoskeleton is variably sclerotised [6] and naturally mineralised with trace elements and metals to provide additional support and toughness [6].

Numerous studies on marine and terrestrial invertebrates have indicated that a variety of trace elements are naturally incorporated into the matrix of the carapace as a means of enhancing the hardness and stiffness of this structure. Trace elements and metals become associated with calcium binding sites of chitin in the exoskeleton of crustaceans such as shrimp and crabs [2,4]. Incorporation of trace elements in the invertebrate carapace is variable between different compartments, with greater mineralisation being observed in areas which are mechanically active [7,8].These include the inner cutting edges of the mandibles of insects [7,9–12] and nereid worms [8,13], the ovipositor in parasitic wasps [14,15] and, tarsal claws and sting of scorpions [7]. It is unclear if the mineralisation of specific body parts and regions of the carapace in crustaceans are similarly variable as in other arthropods.

In addition to natural incorporation of trace elements and metals in the carapace of invertebrates, deposition of metals and trace elements also serves as a means of regulating body burdens [16]. In crustaceans, the fraction of trace elements and metals associated with the exoskeleton accounts for a major portion of the total body burden [2]. Depuration may additionally be associated with direct adsorption from the external surface of the carapace or sequestration of dietary accumulated metals and trace elements within the matrix of the carapace [4]. Periodic moulting of the carapace may therefore serve as an effective means of reducing trace element and metal burdens in the exoskeleton and overall total body concentrations [4]. Studies by Keteles and Fleeger [2], Khan et al. [17] and Wu and Yang [18] found that in several crustaceans, trace element and metals levels in the carapace were higher than in the underlying tissues and organisms collected from polluted locations had higher trace element levels in their carapace than those collected from less polluted sites.

Few investigations have studied the fate of trace elements and metals accumulated by parasites. In endoparasites, such as cestodes and acanthocephalans, Se, Pb and Cd have been associated with the eggshells [19–23]. In the cestodes, *Schyzocotyle acheilognathi* (formally *Bothriocephalus acheilognathi* [24]) and *Bothriocephalus scorpii*, Riggs et al. [19] and Sures et al. [20] showed that Se and, Pb and Cd respectively were more concentrated in the gravid proglottids of the strobila compared to the anterior segments. For the acanthocephalan, *Moniliformis moniliformis*, Scheef et al. [21] found that Cd was more concentrated in female parasites than males, and attributed this to the fact that the metal became sequestered to the eggshells in females. Degger et al. [22] and Kahlil et al. [23] showed that in *S*. *acheilognathi* metals were associated with the eggshells by means of fluorescence microscopy and X-ray microanalysis respectively. In ectoparasites, comparatively fewer studies have investigated trace element depuration in members of this group. Gilbert and Avenant-Oldewage [25] found that in the monogenean, *Paradiplozoon ichthyoxanthon*, trace elements and metals were associated with the vitellaria and sclerites of the parasite, but unlike endoparasite platyhelminthes, the eggshells of this monogenean did not sequester trace elements and metals.

*Argulus japonicus* is a cosmopolitan crustacean ectoparasite displaying low host specificity and therefore infecting a wide range of cyprinid and siluriform fishes. It is most popularly recognised for the devastating effects it has on the aquaculture industry [26]. Until now, little is known about the fate of metals and trace elements in this parasite compared to free-living counterparts and other parasites in general. As with most ectoparasites, trace element and metal bioaccumulation in this parasite still requires investigation. The aims of this study were therefore to assess the trace element and metal sequestration in adult male and female *A*. *japonicus* with fluorescence microscopy. Further comparison between sexes was done to ascertain if sexual dimorphism occurs in *A*. *japonicus* given that male and female parasites have underlying physiological differences. Additionally, the involvement of the eggshell in metal sequestration was assessed to draw parallels between ectoparasites and endoparasites.

## Materials and methods

### Sample collection

Collections of *A*. *japonicus* were conducted in the Vaal Dam, in waters around UJ Island (26°52’33.62”S; 28°10’25.76”E), South Africa. Host cyprinid fishes (*Labeobarbus aeneus*, *Labeobarbus kimberleyensis*, *Labeo capensis* and *Labeo umbratus*) were caught with the aid of gill nets (45–120 mm mesh size) during parasitological surveys in the impoundment. Hosts were removed from the gill nets and macroscopically examined for *A*. *japonicus*, which were removed and placed into plastic specimen jars containing water from the Vaal Dam. The fishes were then placed into an aerated live well and transported back to the UJ Island where they were maintained for further parasitological examinations. Handling of fish was done following approval of the study in accordance with the Ethics Committee of the Faculty of Science, University of Johannesburg (Ethics reference number: 2016-5-03). Argulids were kept in specimen jars until the digestive tract of the parasite was no longer visibly filled with host blood (2–3 days). The parasites were then removed from the jars with the aid of a 3/0 Camel’s hair paintbrush, gender of the parasites was determined and each individual parasite was placed into a separate 2 mL microcentrifuge tube. The tubes were then placed in liquid nitrogen to flash freeze and fix parasites and were later stored at -80 °C in the laboratory until further processing. All collections of host fishes were done following issuing of permits from the Gauteng Department of Agriculture and Rural Development for collection of *L*. *aeneus*, *L*. *capensis* and *L*. *umbratus* (permit number: CPE2–000125), and *L*. *kimberleyensis* (TOPs permit number: 0–09658). As the study was carried out on land belonging to the University of Johannesburg, permission to work from the UJ Island was not required.

### Sample preparation for cryosectioning

A total of 10 *A*. *japonicus* were analysed in the present study. Samples were subdivided for sectioning (n = 4) and whole mounts (n = 6). For sectioning two female and two male parasites were imbedded in optimal cutting temperature (OCT) compound (Sakura Finetek, California, United States of America) and frozen in liquid nitrogen until the compound turned white [25]. The blocks in the moulds were then stored at -80 °C until sectioning. Sections of parasites were prepared at 6 μm with a Reichert–Jung CryoCut E cryomicrotome and mounted on acid washed glass microscope slides. The sections were air dried for 30 minutes to allow adhesion to slides and then stored at -80 °C until staining.

A single adult female *A*. *japonicus* was fixed in acid-formaldehyde-acetic acid and stored in 70% ethanol. This specimen was then dehydrated in acetone and embedded in transmit TAAB resin and sectioned at 5 μm. The sections were then stained with azocaromine-aniline blue (AZAN).

### Fluorescence microscopy

To determine the sites in sections where trace elements were becoming sequestered in parasites, sections and whole mounts were treated with Phen–Green ^TM^ FL cell–permant diacetate (Molecular Probes, Eugene, Oregon, United States of America). This is a broad spectrum fluorochrome for heavy metals such as Cu, Hg, Fe, Pb, Cd and Ni [27]. A 1.5 mM stock solution of Phen–Green was prepared by dissolving 1 mg Phen–Green in 1 mL dimethyl sulphoxide (DMSO) (AppliChem GmbH, Darmstadt, Germany) and from this 10 mL of 0.015 mM working solution was prepared according to Degger et al. [22]. Vials containing solutions were wrapped in tin foil and stored at -20 °C and 4 °C respectively.

Whole mount and sectioned adult parasites were prepared for fluorescence microscopy by first photobleaching the specimens beneath a commercially available ultraviolet lamp (Philips TUV 30W, Holland). For whole mounts two male and two female parasites were photobleached for 20 hours and then treated with Phen–Green. The parasites were mounted on acid washed, glass concavity slides and in the Phen–Green solution, covered with a coverslip and sealed with commercially available clear nail varnish. An additional two adult *A*. *japonicus* (female = 1; male = 1) were not exposed to UV light at room temperature to assess the extent of autofluorescence in the carapace and therefore to assess the effectiveness of photobleaching in reducing interference of autofluorescence. These specimens were mounted on glass concavity slides in diluted DMSO (10% v/v), covered with a coverslip and sealed with clear nail varnish.

Sections of adult parasites were also assessed for autofluorescence. Sections of male and female parasites (n _sections_ = 10 female; 10 male) were treated with 10% DMSO, covered with a coverslip and sealed with clear nail varnish. Sections to be treated with Phen–Green and assessed for autofluorescence were bleached under UV light for 1 hour at room temperature. The sections (n _sections_ = 10 female; 10 male) used for assessing the suitability of the photobleaching procedure were mounted in 10% DMSO and sealed with clear nail varnish. Sections which were treated with Phen–Green were covered with a coverslip and sealed with clear nail varnish. Samples were stored at room temperature for 1 hour in the dark to allow for the fluorochrome to adequately interact with parasite tissue. Sections and whole mounts of *A*. *japonicus* were assessed using a Zeiss Axioplan 2 epi–fluorescence microscope with a fluorescein, rhodamine and DAPI band pass filter (BP 365/12; excitation 490 nm; emission 520 nm). Micrographs were acquired using AxioVision version 4.7.2 software (Carl Zeiss MicroImaging, GmbH, Germany).

### Trace element quantification

Quantification of divalent cations, including trace metals was performed using cryosections of male and female *A*. *japonicus*. The sections were mounted on glass microscope slides and coated with carbon. Coated sections were then assessed using a Tescan Vega 3 scanning electron microscope (Brno, Czech Republic) equipped with a X-Max50 energy-dispersive x-ray spectrometer (Oxford Instruments, Halifax, England), by point analysis at 20 keV operated by Aztec 2.1 software (Oxford Instruments, Halifax England) for Windows. Levels of cations including trace metals were expressed as normalised concentrations in weight percentage (wt%) of specific elements according to the setup of the instrument. As carbon was used to coat the slides and sections, it was included in scans of the sections.

## Results

### Fluorescence microscopy of whole mounts

**Fig 1:**
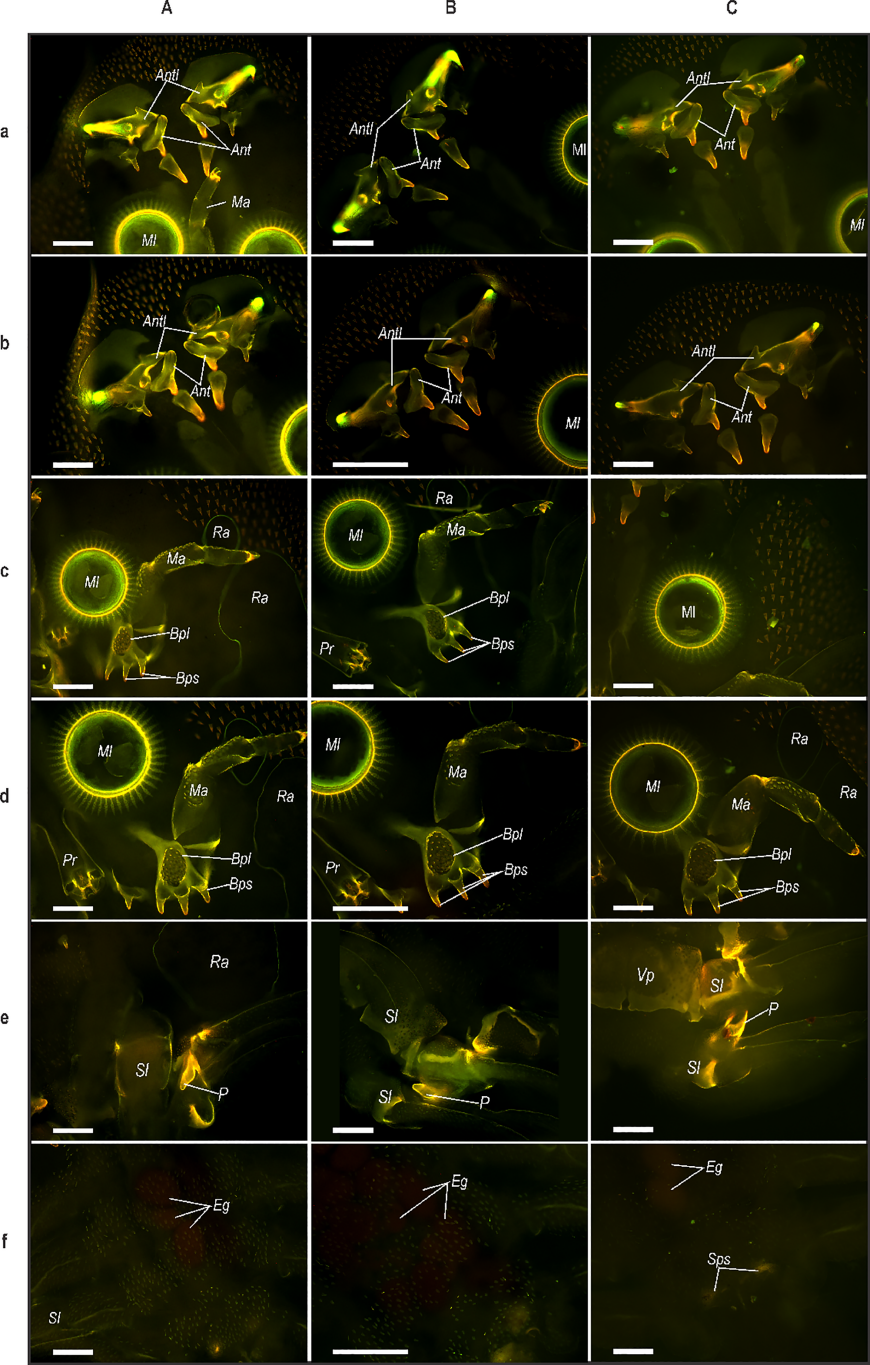
**Whole mounts of male (a; c; e) and female (b; d; f) *Argulus japonicus* showing autofluorescence in unbleached (A) and photobleached (B) specimens, and fluorescence of Phen–Green (C**). (a–d) Micrographs showing the anterior region of adult male and female *A*. *japonicus* where antennules (Antl), antennae (Ant), maxillules (Ml), maxillae (Ma), and basal segment of the maxillae (Bpl) in untreated (A), photobleached (B) and Phen–Green fluorochrome (C). (e–f) Micrographs of the thoracic region of male (e) and female (f) *A*. *japonicus*. In male parasites fluorescence of untreated (A and B) and Phen–Green treated (C) reactions were present in the ventral carapace between the third and fourth swimming legs (Sl) and of the peg (P) of the podomeres of the third pair of swimming legs (Sl). In females the spines on the spermathecae (Sps) were observed for all treatments and *in utero* eggs were observed to produce a positive reaction for the Phen-Green (Cf). All images were taken using Zeiss bandpass filter set 01 (BP 365/12) at 490–520 nm. Scale bars represent 100 μm.

To assess the effectiveness of reducing interference of autofluorescence in whole mounts and sections of adult female and male *A*. *japonicus* photobleached, unbleached and Phen–Green treated specimens were compared (Fig 1). For whole mounts, autofluorescence of the carapace was observed and was reduced after photobleaching. The results show that autofluorescence in both females and males was variable with most of the sclerotised structures showing significantly brighter fluorescence than the rest of carapace. These structures included the antennae and antennules (Fig 1Aa and 1Ab), maxillae and maxillules (Fig 1Ac and 1Ad), spines on the ventral surface of the carapace shield (Fig 1Aa–1Ad) and scales on the ventral surface of the thoracic segments and swimming legs (Fig 1Af). Comparison of autofluorescence signals showed variability between female and male parasites. In males, autofluorescence appeared more intense than in females, particularly for the basal segments of the swimming legs and genital plate associated with the third pair of legs (Fig 1Ae–1Ce). The tips of spines on the distal portions of the antennae and the hooks on the ends of the antennules showed brighter fluorescence than the rest of the structures in female and male parasites (Fig 1Aa–1Ca; 1Ab–1Cb). This was similarly observed for the basal spines on the basal plate of the maxillae (Fig 1Ac and 1Ad). In all of these structures the signal emitted varied from bright orange to yellow to intense green when irradiated using a Zeiss bandpass filter set 01 (BP 365/12). Spines located on the ventral surface of the anterior part of the carapace showed an intense orange–brown colour in both female and male *A*. *japonicus* (Fig 1Aa–1Ad). In whole mounts of female *A*. *japonicus* eggs *in utero* (Fig 1Af and 1Bf) could be seen in bleached and unbleached specimens and produced a red–brown signal. In males, the accessory reproductive structure or peg on the fourth pair of swimming legs showed intense autofluorescence (Fig 1Ae).

Following treatment with the Phen–Green probe positive signals for the fluorochrome were identified in whole mounts of adult parasites (Fig 1Ca–1Cf). Compared to bleached specimens, signals showed variable responses to Phen–Green, which were most intense for structures involved in the attachment, feeding and reproduction. Positive fluorescence was identified for the antennules (Fig 1Ca and 1Cb), specifically in the area where the structure terminated in a hook. The spines located on the ventral surface of the carapace similarly showed a positive response to the fluorochrome, with most intense signals being identified for the distal region of the structure. In the maxillules (Fig 1Cc and 1Cd), rings of sclerotised tissue and supportive bars on the distal surface reacted positively to the probe. Differences in reactions in males and females were specifically identified for the thorax and reproductive areas associated with appendages in this region (Fig 1Ce and Cf). The ventral surface of both male (Fig 1Ce) and female (Fig 1Cf) *A*. *japonicus* are adorned with scales which showed brighter fluorescence than the carapace beneath these. Furthermore, these are located in “bands” which spread between the pairs of swimming legs on each segment. Generally in male parasites, the signal produced by ornamentation structures was more intense in Phen–Green specimens than in female specimens (Fig 1Ca–1Cf). In whole mounts of female parasites, *in utero* eggs (Fig 1Cf) were observed to have produced a light orange–brown signal in contrast to the red–brown signal observed in untreated specimens. In whole mounts of male *A*. *japonicus* treated with Phen–Green, the peg (Fig 1Ce) on the third pair of swimming legs and basal plate/genital plate of the carapace showed intense yellow fluorescence when treated with the Phen–Green probe (Fig 1Ce). This was much brighter than the signal observed for the rest of the carapace in this region.

### Fluorescence microscopy in cryosections

Following from the results observed for autofluorescence in whole mounts of adult male and female *A*. *japonicus*, sections of adult parasites indicated that autofluorescence was mostly related to the sclerotised structures of the carapace, such as the antennules, hooks and scales present on the ventral surface of the carapace rather than internal organs and soft tissues. Comparison of sections which were photobleached and unbleached with sections treated with Phen–Green, signals were more intense for the fluorescent probe than in untreated sections. Therefore, treatment with Phen–Green indicated positive green–yellow signals for divalent cations which included trace metals in the cuticle and internal tissues for both male and female *A*. *japonicus*.

**Fig 2:**
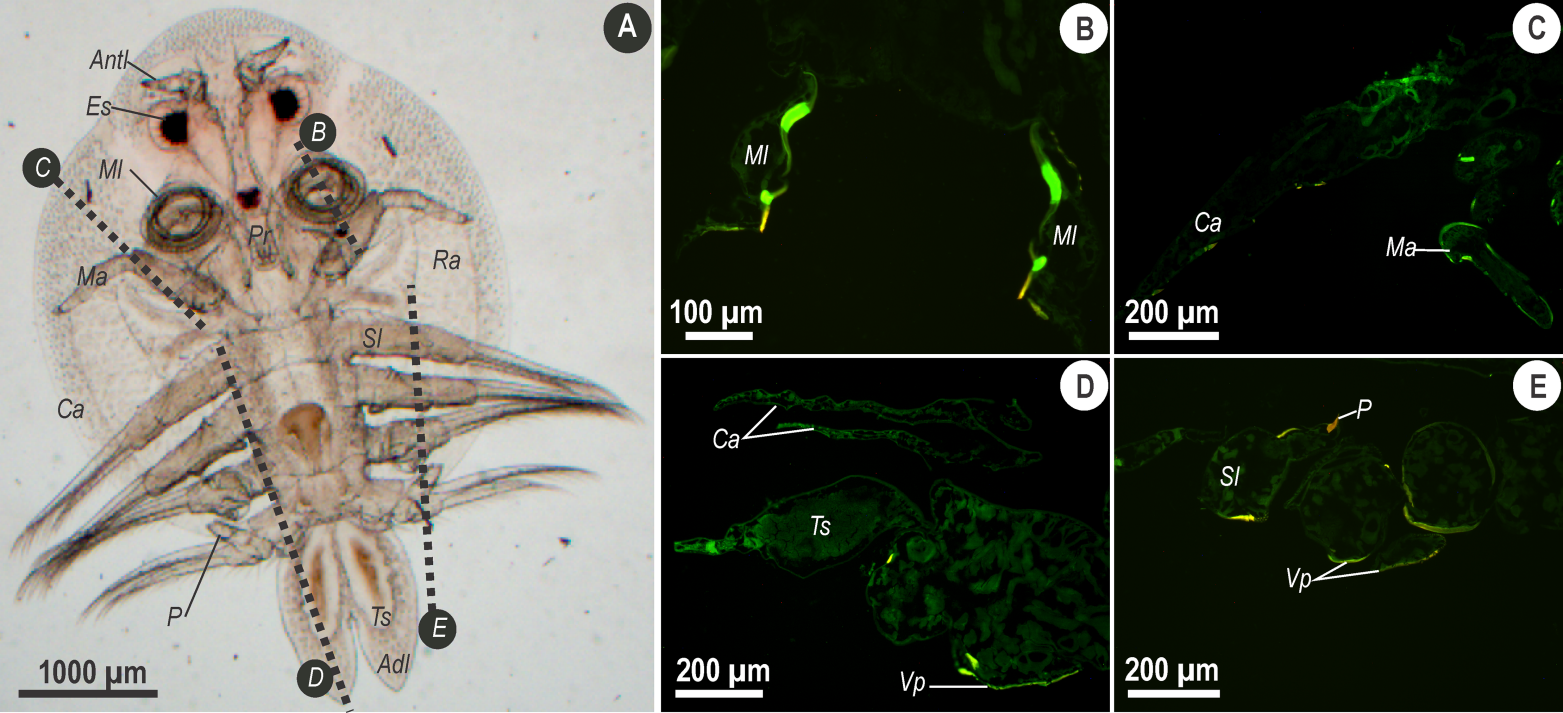
**Micrographs showing whole mount (A) and longitudinal sections (B, C, D and E) through male *Argulus japonicus*.** Section planes are indicated on the light micrograph of a whole mount of a male *A*. *japonicus* (A) with dotted lines indicating sections for B–E. Fluorescence of the Phen–Green probe indicates a positive reaction for divalent cations including trace metals in longitudinal sections (B–C) of the maxillule (Ml) and maxilla (Ma). The cuticle of these regions produce more intense signals than the cuticle of the carapace (Ca) of the cephalic shield. (D) A longitudinal section through the posterior region of the thorax and abdomen shows positive fluorescence for the carapace (Ca) of the lobes of cephalic shield and the testes (Ts) in the abdomen. More intense signal was observed for the portion of the carapace of the ventral side of the thorax (Vp). (E) Longitudinal section through the thorax shows positive signals for the peg (P) of the third pair of swimming legs (Sl) and the carapace of the ventral surface of the thorax (Vp). Images were acquired using Zeiss bandpass filter set 01 (BP 365/12) at 490–520 nm.

In Fig 2, results of Phen–Green treated sections of male *A*. *japonicus* are shown. A light micrograph of a male parasite (Fig 1A) has been included for purposes of orienting where sections were taken. Sections through the maxillule (Fig 2B) confirmed the findings in whole mounts where two distinct bands within these structures could be seen. Following treatment with Phen–Green, the carapace of the cephalic shield produced a green signal which was brighter than the signal produced for internal tissues (Fig 2C and 2D). In addition, fluorescent signals produced for the ventral carapace were more intense than other areas of the carapace (Fig 2D and 2E). Section through the posterior region of the thorax (Fig 2E) showed that the signal associated with the peg was distinct and more intense compared to the ventral portions respectively. The peg could be seen as producing an intense orange signal compared to the bright yellow signal of the cuticle.

**Fig 3:**
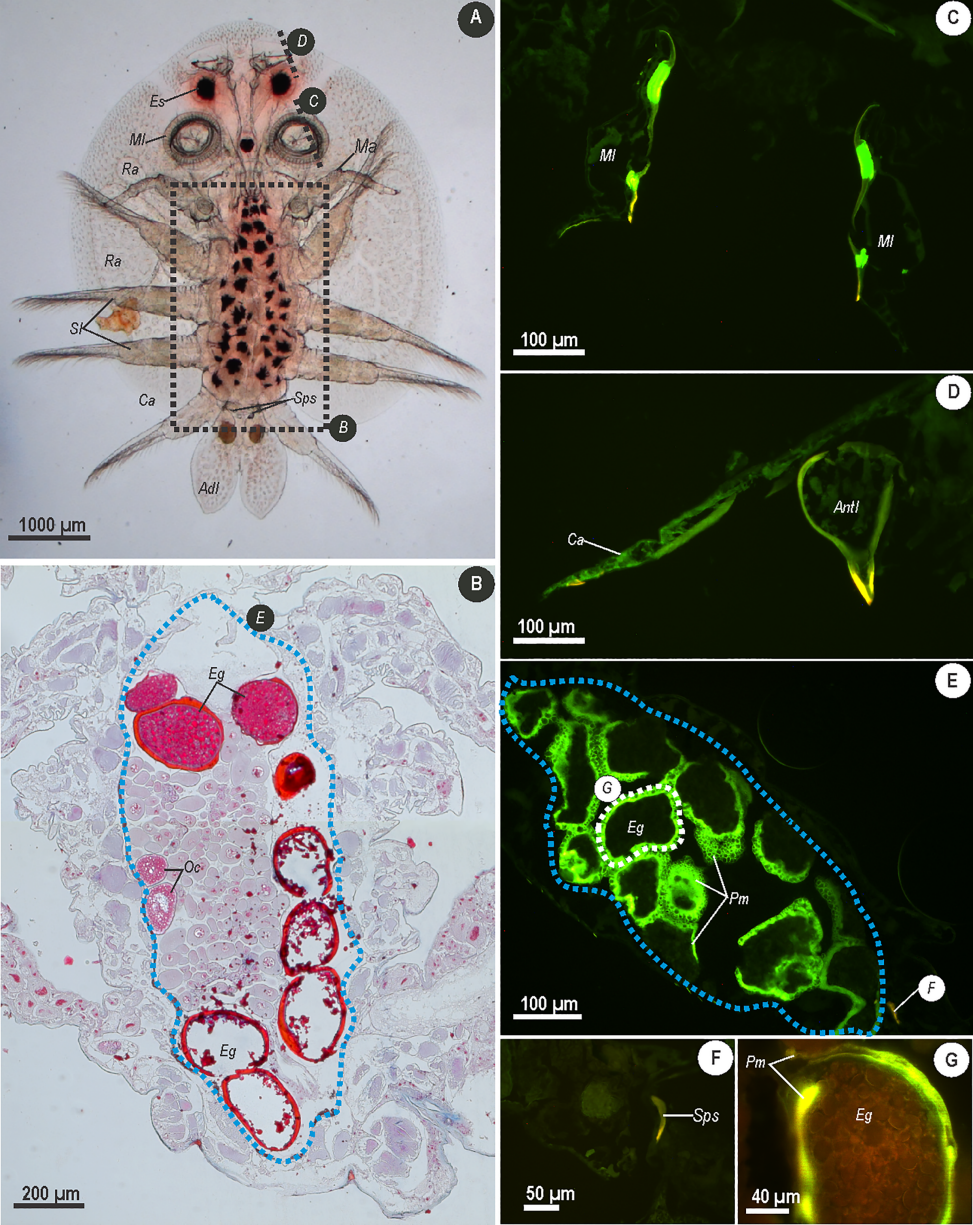
**Micrographs showing whole mount (A), frontal section (B) stained with Azocarmine–Aniline blue (AZAN) and longitudinal sections treated with Phen–Green (C, D, E, F and G) through female *Argulus japonicus*.** (A) Section planes are indicated on the light micrograph of an adult female *A*. *japonicus* with dotted lines for sections C and D; dotted line indicated labelled B indicates area where frontal section of the reproductive tract imaged in image B. (B) Frontal section of the thorax stained with AZAN containing mature oocytes (Oc) and shelled eggs (Eg); blue dotted line E corresponds with fluorescence micrograph E. (C) Section through the maxillule (Ml) of treated with Phen–Green. (D) Longitudinal section through the cephalic shield of the carapace (Ca) and antennule (Antl). (E) Longitudinal section through the thorax treated with Phen–Green showing fluorescence amorphous layer (Pm) surrounding eggs (Eg) *in utero* which corresponds with image G. (F) Section through the spermathaceal spine (Sps) treated with Phen–Green. (G) Section through an *in utero* egg (Eg) showing intense signal produced by amorphous layer (Pm) surround the egg. Images were acquired using Zeiss bandpass filter set 01 (BP 365/12) at 490–520 nm.

As presented for male *A*. *japonicus*, in females a light micrograph (Fig 3A) has been included to assist in orientation of structures and sections indicated in fluorescence images (Fig 3C–3G). Additionally, a light micrograph of a frontal section through the thorax of a female parasite (Fig 3B) serves to indicate histological features associated with the reproductive system.

Fluorescence signals in female *A*. *japonicus* were generally weaker compared to sections through male parasites for the general cuticle. However, variability in the signals produced in specific structures were similar. For instance sections through the maxillules (Fig 3C) and antennules (Fig 3D) produced similarly intense signals compared to males. As in males, the maxillules (Fig 3C) of females showed positive fluorescence for two distinct bands within this structure. Similarly, a section through the antennule (Fig 3D) shows that the signal of the fluorochrome becomes more intense toward the hooked portion of the structure. In females, although *in utero* eggs in whole mounts treated with Phen–Green showed a weak orange–brown signal, sections through the thorax showed a layer surrounding the eggs which produced a bright and intense green signal (Fig 3E). The identification of this layer was confirmed in female specimens of *A*. *japonicus* stained with AZAN (Fig 3B). At higher magnification, the shell of the egg was distinguishable and produced an orange signal. Surrounding the shell, the amorphous layer produced an intense yellow–green signal (Fig 3G). In addition to the fluorescence of the eggs, the spermathecal spines were identifiable in sections and produced a weak yellow signal (Fig 3E and 3F).

### EDS analysis of cryosections

**Fig 4:**
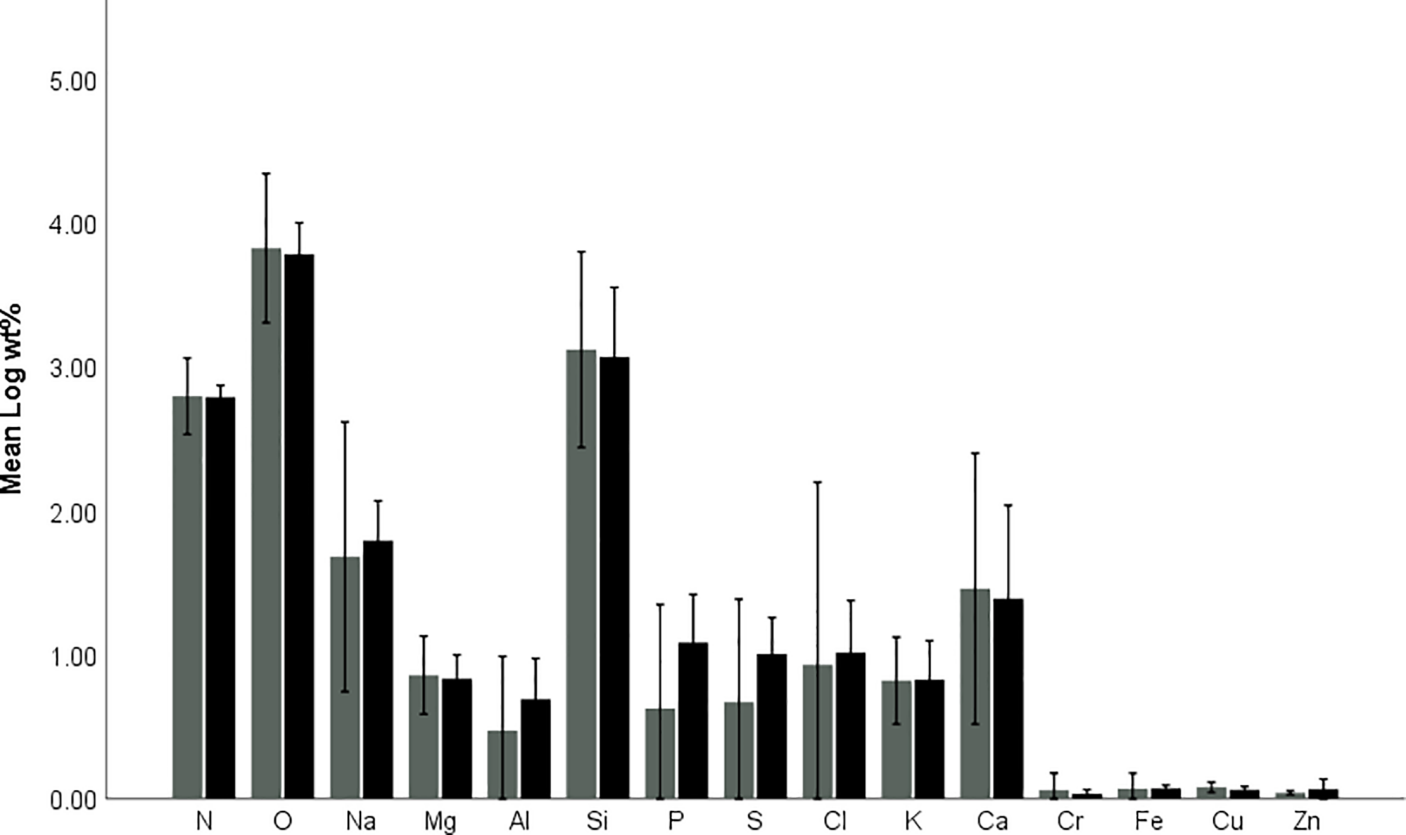
Bar graph showing means for the natural logarithm of the weight percentages (wt%) for elements identified in sections of male (black bars) and female (grey bars) *Argulus japonicus* by SEM/EDS. Error bars indicate 95% confidence intervals.

As a means of identifying elements in sections of adult parasites which correspond to fluorescence observed in different structures in male and female *A*. *japonicus* was performed using SEM/EDS (Fig 4). Results of the analysis showed trace amounts of N, O, Na, Mg, Al, Cl, K, Ca, Cr, Fe, Cu and Zn were present in the carapace of male and female *A*. *japonicus*. Differences in weight percentage of the elements detected were not different between male and female parasite (Mann-Whitney-U test, Z = -0.441; p = 0.659). Similarly, comparison between element levels in the structures analysed from male and female parasites showed that levels were not significantly different (Kruskall-Wallis, male: *x*^*2*^ = 0.129; *df* = 4; p = 0.998; female: *x*^*2*^ = 0.636; *df* = 2; p = 0.727).

## Discussion

In the present study, the use of fluorescence microscopy as a tool for studying the mineralisation and incorporation of divalent cations, which include trace elements and metals, in the carapace of the ectoparasite, *Argulus japonicus*, was assessed. Comparison of the results of autofluorescence of male and female parasites to bleached and stained specimens indicated that certain features of the carapace are more reactive with the fluorescent light and Phen–Green probe respectively. The use of fluorescence microscopy in the present study not only allowed for the discernment of trace element and metal accumulation in *A*. *japonicus* but also serves to indicate the validity of the technique for studying a number of morphological characters of crustacean parasites. Analysis of hard structures in ectoparasites has been attempted and similarly found useful for discerning characteristics which are otherwise not possible to study with conventional microscopy [28–30]. Haug et al. [28] showed that more sclerotised regions of the carapace of *A*. *foliaceus* and other crustaceans, such as *Limulus polyphemus* and *Anaspides tasmaniae*, produced brighter autofluorecent signals than less sclerotised areas of the carapace. Additionally, Milne and Avenant-Oldewage [29] and Wong et al. [30] showed that fluorescence of the haptoral sclerites of monogenean parasites, *Paradiplozoon ichthyoxanthon* and *Diplozoon paradoxum* respectively could be used to study the morphology of these structures. In addition to observing clear morphological characteristics in adult male and female *A*. *japonicus*, dimorphism between sexes was further observed in varying degrees of sclerotization of features of the carapace [28,31]. Appendages principally used in attachment of parasites to hosts were observed to be more sclerotised than areas of the carapace which were not involved in this function. Areas of the carapace, which were more sclerotised, showed brighter autofluorescence and agree with findings of Gresty et al. [31]. These results similarly corroborate the work of Haug et al. [28] who also found that more sclerotised structures of the crustacean carapace produced more intense autofluorescence. Additionally, the bright signal produced for these structures following treatment with Phen–Green additionally suggest that trace element and metals are concentrated in these areas of the carapace compared to the rest of the carapace.

Reaction of the Phen–Green probe in whole mounts and sections of *A*. *japonicus* indicated positive results for the incorporation of trace elements and metals in the matrix of the carapace. Following bleaching of whole mounts and sections, treatment of specimens with the fluorochrome further indicated that although metals are incorporated into the entire carapace, mineralisation appears to be more pronounced in areas where sclerotization is more prominent. Areas which showed intense autofluorescence also showed more intense fluorescence for Phen–Green than areas where the cuticle is less sclerotised. Higher concentrations of trace elements and metals have been found to be associated with more sclerotised areas of the carapace in other aquatic and terrestrial invertebrates of mechanically active appendages. This was shown for the cutting edges of mandibles of leaf-cutter ants, *Atta sexdens rubipilosa* [10,32] and *Nereis virens* [13], stings and tarsal claws of scorpions, *Vaejovis spinigeris* and *Centruroides exilicauda* [7], and ovipositor of the parasitic wasp, *Schlettereriella* sp. and other hymenopterous insects [14,33]. In most instances, Zn has been found to be the dominant element present in areas where trace element mineralisation has occurred in the carapace. Edwards et al. [32] found that Zn was localised in the margins of the cutting edges of the cuticle of mandibles in the leaf cutter ant, *Atta sexedens rubropilosa*. This was similarly observed by Schofield et al. [7,10], Cribb et al. [11], Lichtenegger et al. [8] and Broomell et al. [13] in the mandibles of ants, termites and polychaete worms respectively. In addition, Zn, Cu and Mn have also been identified in the cuticles of invertebrates [7,8,12,13]. Although numerous metals have the potential to bind in areas of the cuticle, some are not involved in providing mechanical support to organs [34]. Cribb et al. [11,12] suggested that although Mn was incorporated into “tool” structures of insects it was not related to enhancement of hardness and stiffness of the cuticle, but rather was suggested to provide support to mechanical properties of the cuticle. In *A*. *japonicus*, incorporation of trace elements and metals into the carapace, specifically in areas which function in direct attachment (eg. antennae and maxillules) or assisted attachment (eg. spines and scales) to hosts would have to be strong enough to withstand the force of water passing over the surface of the fish, particularly if hosts were swimming at high speed. In this way strengthening of these organs would ensure the carapace would be strong enough to withstand damage from water passing over the parasite rapidly or becoming dislodged from hosts as they rub against coarse substrata and abrasive objects in an attempt to dislodge parasites.

Results of SEM/EDS analysis of the levels of trace elements including metals in cryosections of male and female *A*. *japonicus* showed that N, O, Na, Mg, Al, Cl, K, Ca, Cr, Fe, Cu and Zn are present. In particular, elements such as Al, Cr, Fe, Cu and Zn become incorporated in the carapace, which is similar to other marine and terrestrial invertebrates [7,8,10,12,13] and hard structures as in other ectoparasites [25]. Furthermore, this shows that *A*. *japonicus* do accumulate trace elements which are present in the macroenvironment but further study into the bioaccumulation capacity of these parasites is required to properly understand this. In both male and female parasites varying degrees of mineralisation in the cuticle of the cephalic shield and covering the ventral surface of the body was found. Such differential mineralisation in these areas of the carapace indicate that the cephalic shield is softer and more malleable than the cuticle on the ventral side of the body, which is more rigid. Such findings are in line with the general function of these areas of the cuticle. The cephalic shield functions in enabling the parasite to move effectively through the water column, by manipulating the position and shape of the dorsal cuticle. Parasites are, therefore, able to effectively change direction and actively swim in search for hosts. Greater mineralisation of the cuticle covering the thoracic segments would be necessary for providing protection to the sensitive organs within this region of both male and female parasites. Differences in concentrations of chitin in several male and female grasshopper species were indicated by Kaya et al. [35], with male grasshoppers having higher chitin levels and thicker chitin nanofibers than in female grasshoppers. Weaker fluorescence of the Phen–Green probe in female *A*. *japonicus* could further point to less chitin being present in the carapace and as a result fewer binding locations for trace elements and metals would be present. In female parasites a less mineralised and softer cuticle would allow for greater expansion of the thorax in order to accommodate large numbers of developing eggs.

Sexual dimorphic differences in the mineralisation of the carapace of male and female *A*. *japonicus* were also identified, with males generally displaying stronger reactions of the Phen–Green probe than females. This was particularly noticeable for the carapace surrounding the thoracic segments. In addition, the intense reaction of the cuticle of the peg structure on the fourth pair of swimming legs in unbleached and Phen–Green treated parasites with fluorescent light indicated that this structure is sclerotised and mineralised. The peg is important as it is utilised by male parasites for transfer of spermatophores to female parasites [36]. In females, the fluorescence of the spermathecal spines located beneath the natatory lobes indicated that these structures are similarly mineralised with trace elements and metals. Hardening through mineralisation of the cuticle lining these structures would be necessary as the spines are used to inseminate each individual egg [36] as they pass through the gonopore during laying. In this way, hardening of these structures would enhance reproductive success. Sclerotisation of reproductive organs have similarly been found in the ovipositors of parasitic wasps, which are used to deposit fertilised eggs when either directly or indirectly penetrating the hard cuticle of potential invertebrate hosts [14,15,33]. Ghara et al. [15] further indicated that the degree of sclerotization between parasitic wasps differed and was related to the method of oviposition. Highly mineralised ovipositors in parasitoids were necessary as oviposition was achieved externally through the wall of the fig syconium, whereas, unmineralised variants in gallers were related to oviposition directly through the stigma of the internal surface of the syconium.

Along with the positive role of mineralising specific elements on the carapace in *A*. *japonicus* in terms of attachment and support of attachment structures, the deposition of excess trace elements and metals in the carapace may additionally serve a regulatory pathway. The function of the carapace in reducing body burdens of trace elements and metals has been studied and shown for numerous free-living crustaceans [2,4,37]. During ecdysis or cuticle shedding, the excess metals sequestered and adsorbed to the carapace are released from the body to the environment [2,37]. Keteles and Fleegar [2] found that shedding of the carapace during ecdysis was effective in reducing Cd burdens in grass shrimp (*Palaeminetes pugio*), and Bergey and Weis [4] found elevated levels of Cu, Pb and Zn in the carapace of fiddler crabs (*Uca pugnax*) molted during ecdysis with levels significantly higher in crabs from polluted areas compared to unpolluted sites. Wu and Yang [18] found that Cr and Mn were concentrated in the carapace of white shrimp (*Litopenaeus vannamei*) compared to other tissues analysed. Khan et al. [17] found that metals accumulated by *Gammarus pulex* first became bound as metal rich granules which were then sequestered into the carapace and once fed to zebrafish (*Danio rerio*) pronounced lipid peroxidation in the gut of fish was observed and associated with elevated metal concentrations in the carapace of *G*. *pulex*. In *A*. *japonicus* it is likely that high burdens of elements are reduced in a similar fashion but this also appears to vary between female and male parasites.

The intense green fluorescence of the amorphous material which surrounds the eggs and provides a means of securing eggs to substratum once laid by females may also function in releasing trace elements and metals from the parasite. Maternal transfer of trace elements and metals, such as Se, as a mode of reducing body burdens in female invertebrates has been indicated in mayflies, *Centroptilum triangulifer* [38] and some endoparasites [19–23]. In cestodes, *S*. *acheilognathi* and *B*. *scorpii*, Riggs et al. [19] and Sures et al. [20] found that levels of Se and, Cd and Pb respectively were higher in the posterior, gravid proglottids of the strobila than in the anterior segments. Scheef et al. [21] found that female acanthocephalan (*M*. *moniliformis*) had higher burdens of Cd than males and related this to the fact that the metal became bound to the eggshell. Degger et al. [22] and Khalil et al. [23] both showed that metals are associated with the eggshells of the *S*. *acheilognathi* through use of fluorescence microscopy and x-ray microanalysis respectively. Differences between the accumulation of trace elements and metals in male and female *A*. *japonicus* in the present study are therefore, speculative as no formal quantification of concentrations of these elements was conducted, however, in *A*. *japonicus* it would appear that eggshells do not function in a similar manner given the negative signal produced by the eggshell (Fig 3G). Instead, the findings of the present study are similar to those observed for the monogenean *Paradiplozoon ichthyoxanthon* treated with Phen–Green where similar orange signal was observed and indicated a negative reaction for the presence of metals in eggshells [25]. Degger et al. [22] indicated that trace element and metal sequestration in the eggshells of *S*. *acheilognathi* was related to age with younger eggs showing an orange signal which was negative for metals. The incorporation of metals into the shell matrix is believed to occur during the tanning of the quinone residues by enzymatic activity. As indicated by Gilbert and Avenant-Oldewage [25], a negative signal for trace elements and metals in the shells of *P*. *ichthyoxanthon* could be related to the lack of quinones in the eggshells [39,40], which are likely composed of dityrosine and hardened through dehydration of the shell matrix and not through enzymatic polymerisation [40]. Formation of the eggshell in *A*. *japonicus* has been shown to occur directly from the oocyte where two distinct layers have been observed [41]. Given the parallels between the fluorescent results in eggs of *P*. *ichthyoxanthon* presented by Gilbert and Avenant-Oldewage [25] and the present results for eggshells of *A*. *japonicus* it is likely that the lack of trace elements and metals in the eggshells could result from similar processes of eggshell hardening in the latter. As for the amorphous layer surrounding the eggs in female *A*. *japonicus*, positive intense green signals for trace elements and metals following treatment with Phen–Green further suggests a route through which female parasites are able to regulate trace element burdens. Shafir and van As [42] reported that this layer is composed of a gelatinous material which hardens when coming in contact with the aquatic environment and functions in securing the eggs to a substratum. The exact biochemistry of this material has yet to be described, but from the current results of female parasite sections stained with the trichrome AZAN, it is possibly proteinaceous in nature. A similar phenomenon has been described by Fryer [43] in the spermatophore of *Dolops ranarum*, once secured to the female parasite by a male, the outer surface of the spermatophore hardens. Fryer [43] suggested that this layer is composed of a proteinaceous material produced by spermatophore glands. Given the similarity in nature of these layers, the biochemistry of the material in eggs of *A*. *japonicus* may be similar, however, further studies are required to ascertain the biochemistry of this material.

## References

[1] Rainbow PS. Trace metal concentrations in aquatic invertebrates: why and so what? Environ Pollut. 2002;120: 497–507. doi: 10.1016/S0269-7491(02)00238-5 12442773 10.1016/s0269-7491(02)00238-5

[2] Keteles KA, Fleeger JW. The contribution of ecdysis to the fate of copper, zinc and cadmium in grass shrimp, *Palaemonetes pugio* Holthius. Mar Pollut Bull. 2001;42: 1397–1402 11827128 10.1016/s0025-326x(01)00172-2

[3] Ahearn GA, Mandal PK, Mandal A. Mechanisms of heavy-metal sequestration and detoxification in crustaceans: A review. J Comp Physiol B Biochem Syst Environ Physiol. 2004;174: 439–452. doi: 10.1007/s00360-004-0438-0 15243714 10.1007/s00360-004-0438-0

[4] Bergey LL, Weis JS. Molting as a mechanism of depuration of metals in the fiddler crab, *Uca pugnax*. Mar Environ Res. 2007;64: 556–562. doi: 10.1016/j.marenvres.2007.04.009 17590429 10.1016/j.marenvres.2007.04.009

[5] Jonusaite S, Donini A, Kelly SP. Occluding junctions of invertebrate epithelia. J Comp Physiol B Biochem Syst Environ Physiol. 2016;186: 17–43. doi: 10.1007/s00360-015-0937-1 26510419 10.1007/s00360-015-0937-1

[6] Melnick CA, Chen Z, Mecholsky JJJ. Hardness and toughness of exoskeleton material in the stone crab, *Menippe mercenaria*. J Mater Res. 1996;11: 2903–2907. doi: 10.1557/JMR.1996.0367

[7] Schofield RMS, Nesson MH, Richardson KA, Wyeth P. Zinc is incorporated into cuticular “tools” after ecdysis: The time course of the zinc distribution in “tools” and whole bodies of an ant and a scorpion. J Insect Physiol. 2003;49: 31–44. doi: 10.1016/S0022-1910(02)00224-X 12770014 10.1016/s0022-1910(02)00224-x

[8] Lichtenegger HC, Schöberl T, Ruokolainen JT, Cross JO, Heald SM, Birkedal H, et al Zinc and mechanical prowess in the jaws of *Nereis*, a marine worm. Proc Natl Acad Sci. 2003;100: 9144–9149. doi: 10.1073/pnas.1632658100 12886017 10.1073/pnas.1632658100 PMC170886

[9] Hillerton JE, Robertson B, Vincent JFV. The presence of zinc or manganese as the predominant metal in the mandibles of adult, stored-product beetles. J Stored Prod Res. 1984;20: 133–137. doi: 10.1016/0022-474X(84)90020-1

[10] Schofield RMS, Nesson MH, Richardson KA. Tooth hardness increases with zinc-content in mandibles of young adult leaf-cutter ants. Naturwissenschaften. 2002;89: 579–583. doi: 10.1007/s00114-002-0381-4 12536282 10.1007/s00114-002-0381-4

[11] Cribb BW, Stewart A, Huang H, Truss R, Noller B, Rasch R, et al Insect mandibles—comparative mechanical properties and links with metal incorporation. Naturwissenschaften. 2008;95: 17–23. doi: 10.1007/s00114-007-0288-1 17646951 10.1007/s00114-007-0288-1

[12] Cribb BW, Lin CL, Rintoul L, Rasch R, Hasenpusch J, Huang H. Hardness in arthropod exoskeletons in the absence of transition metals. Acta Biomater. 2010;6: 3152–3156. doi: 10.1016/j.actbio.2010.02.009 20152944 10.1016/j.actbio.2010.02.009

[13] Broomell CC, Zok FW, Waite JH. Role of transition metals in sclerotization of biological tissue. Acta Biomater. 2008;4: 2045–2051. doi: 10.1016/j.actbio.2008.06.017 18653388 10.1016/j.actbio.2008.06.017

[14] Quicke DLJ, Wyeth P, Fawke JD, Basibuyuk HH, Vincent JFV. Manganese and zinc in the ovipositors and mandibles of hymenoptereous insects. Zool J Linn Soc. 1998;124: 387–396.

[15] Ghara M, Kundanati L, Borges RM. Nature’s swiss army knives: Ovipositor structure mirrors ecology in a multitrophic fig wasp community. PLoS One. 2011;6: e23642 doi: 10.1371/journal.pone.0023642 21909352 10.1371/journal.pone.0023642 PMC3166121

[16] Raessler M, Rothe J, Hilke I. Accurate determination of Cd, Cr, Cu and Ni in woodlice and their skins—is moulting a means of detoxification? Sci Total Environ. 2005;337: 83–90. doi: 10.1016/j.scitotenv.2004.07.008 15626381 10.1016/j.scitotenv.2004.07.008

[17] Khan FR, Bury NR, Hogstrand C. Cadmium bound to metal rich granules and exoskeleton from *Gammarus pulex* causes increased gut lipid peroxidation in zebrafish following single dietary exposure. Aquat Toxicol. 2010;96: 124–129. doi: 10.1016/j.aquatox.2009.10.010 19883947 10.1016/j.aquatox.2009.10.010

[18] Wu X-Y, Yang Y-F. Heavy metal (Pb, Co, Cd, Cr, Cu, Fe, Mn and Zn) concentrations in harvest-size white shrimp *Litopenaeus vannamei* tissues from aquaculture and wild source. J Food Compos Anal. 2011;24: 62–65. doi: 10.1016/j.jfca.2010.03.030

[19] Riggs MR, Lemly AD, Esch GW. The growth, biomass, and fecundity of *Bothriocephalus acheilognathi* in a North Carolina cooling reservoir. J Parasitol. 1987;73: 893–900. 3656010

[20] Sures B, Taraschewski H, Rokicki J. Lead and cadmium content of two cestodes, *Monobothrium wageneri* and *Bothriocephalus scorpii* and their fish hosts. Parasitol Res. 1997;83: 618–623. doi: 10.1007/s004360050307 9211516 10.1007/s004360050307

[21] Scheef G, Sures B, Taraschewski H. Cadmium accumulation in *Moniliformis moniliformis* (Acanthocephala) from experimentally infected rats. Parasitol Res. 2000;86: 688–691. doi: 10.1007/PL00008553 10952271 10.1007/pl00008553

[22] Degger N, Avenant-Oldewage A, Greenfield R. Innovative fluorescence detection technique for metals in cestode egg-shells. African Zool. 2009;44: 204–207. doi: 10.3377/004.044.0208

[23] Khalil M, Furness D, Polwart A, Hoole D. X-ray microanalysis (EDXMA) of cadmium-exposed eggs of *Bothriocephalus acheilognathi* (Cestoda: Bothriocephalidea) and the influence of this heavy metal on coracidial hatching and activity. Int J Parasitol. 2009;39: 1093–1098. doi: 10.1016/j.ijpara.2009.02.023 19341741 10.1016/j.ijpara.2009.02.023

[24] Brabec J, Waeschenbach A, Scholz T, Littlewood DTJ, Kuchta R. Molecular phylogeny of the Bothriocephalidea (Cestoda): Molecular data challenge morphological classification. Int J Parasitol. 2015;45: 761–771. doi: 10.1016/j.ijpara.2015.05.006 26183667 10.1016/j.ijpara.2015.05.006

[25] Gilbert BM, Avenant-Oldewage A. Metal sequestration in vitellaria and sclerites, and reactive oxygen intermediates in a freshwater monogenean, *Paradiplozoon ichthyoxanthon*. PLoS One. 2017;12: e0177558 doi: 10.1371/journal.pone.0177558 28498876 10.1371/journal.pone.0177558 PMC5428946

[26] Alsarakibi M, Wadeh H, Li G. Parasitism of *Argulus japonicus* in cultured and wild fish of Guangdong, China with new record of three hosts. Parasitol Res. 2014;113: 769–775. doi: 10.1007/s00436-013-3708-5 24297692 10.1007/s00436-013-3708-5

[27] Hauglund RP. The Handbook-A guide to fluorescent probes and labelling technologies. 10th Editi. Eugene, USA: Invitrogen Corp.; 2005.

[28] Haug JT, Haug C, Kutschera V, Mayer G, Maas A, Liebau S, et al Autofluorescence imaging, an excellent tool for comparative morphology. J Microsc. 2011;244: 259–272. doi: 10.1111/j.1365-2818.2011.03534.x 21883208 10.1111/j.1365-2818.2011.03534.x

[29] Milne SJ, Avenant-Oldewage A. The fluorescent detection of *Paradiplozoon* sp. (Monogenea: Diplozoidae) attachment clamps’ sclerites and integumental proteins. Onderstepoort J Vet Res. 2006;73: 149–52. 16958267 10.4102/ojvr.v73i2.161

[30] Wong WL, Michels J, Gorb SN. Resilin-like protein in the clamp sclerites of the gill monogenean *Diplozoon paradoxum* Nordmann, 1832. Parasitology. 2012; 1–4. doi: 10.1017/S0031182012001370 22939032 10.1017/S0031182012001370

[31] Gresty KA, Boxshall GA, Nagasawa K. The fine structure and function of the cephalic appendages of the branchiuran parasite, *Argulus japonicus* Thiele. Philos Trans R Soc London B. 1993;339: 119–135.

[32] Edwards AJ, Fawke JD, McClements JG, Smith SA, Wyeth P. Correlation of zinc distribution and enhanced hardness in the mandibular cuticle of the leaf-cutting ant *Atta sexdens rubropilosa*. Cell Biol Int. 1993;17: 697–698. doi: 10.1006/cbir.1993.1125

[33] Quicke DLJ, Palmer-Wilson J, Burrough A, Broad GR. Discovery of calcium enrichment in cutting teeth of parasitic wasp ovipositors (Hymenoptera: Ichneumonoidea). African Entomol. 2004;12: 259–264.

[34] Morgan TD, Baker P, Kramer KJ, Basibuyuk HH, Quicke DLJ. Metals in mandibles of stored product insects: do zinc and manganese enhance the ability of larvae to infest seeds? J Stored Prod Res. 2003;39: 65–75. doi: 10.1016/S0022-474X(02)00019-X

[35] Kaya M, Lelešius E, Nagrockaitė R, Sargin I, Arslan G, Mol A, et al Differentiations of chitin content and surface morphologies of chitins extracted from male and female grasshopper species. PLoS One. 2015;10: e0115531 doi: 10.1371/journal.pone.0115531 25635814 10.1371/journal.pone.0115531 PMC4312026

[36] Avenant-Oldewage A, Everts L. *Argulus japonicus*: Sperm transfer by means of a spermatophore on *Carassius auratus* (L). Exp Parasitol. 2010;126: 232–238. doi: 10.1016/j.exppara.2010.05.011 20493846 10.1016/j.exppara.2010.05.011

[37] Robinson KA, Baird DJ, Wrona FJ. Surface metal adsorption on zooplankton carapaces: implications for exposure and effects in consumer organisms. Environ Pollut. 2003;122: 159–167. doi: 10.1016/S0269-7491(02)00302-0 12531303 10.1016/s0269-7491(02)00302-0

[38] Conley JM, Funk DH, Buchwalter DB. Selenium bioaccumulation and maternal transfer in the mayfly *Centroptilum triangulifer* in a life-cycle, periphyton-biofilm trophic assay. Environ Sci Technol. 2009;43: 7952–7957. doi: 10.1021/es9016377 19921919 10.1021/es9016377

[39] Ramalingam K. Chemical nature of the egg shell in helminths: II. Mode of stabilization of egg shells on monogenetic trematodes. Exp Parasitol. 1973;34: 115–122. 4124670 10.1016/0014-4894(73)90069-6

[40] Wharton DA. The production and functional morphology of helminth egg-shells. Parasitology. 1983;86: 85–97. 6346235 10.1017/s003118200005085x

[41] Ikuta K, Makioka T, Amikura R. Eggshell ultrastructure in *Argulus japonicus* (Branchiura). J Crustac Biol. 1997;17: 45–51.

[42] Shafir A, Van As JG. Laying, development and hatching of eggs of the fish ectoparasite *Argulus japonicus* (Crustacea: Branchiura). J Zool Lond. 1986;210: 401–414.

[43] Fryer G. The spermatophores of *Dolops ranarum* (Crustacea, Branchiura): their structure, formation and transfer. Q J Microsc Sci. 1960;101: 407–432.

